# Effects of Different Recovery Duration on External and Internal Load Measures during Bouts of Small-Sided Games

**DOI:** 10.5114/jhk/169520

**Published:** 2023-10-11

**Authors:** Vicente de Dios-Álvarez, Alexis Padrón-Cabo, Miguel Lorenzo-Martínez, Ezequiel Rey

**Affiliations:** 1 Faculty of Education and Sport Sciences, University of Vigo, Pontevedra, Spain.; 2 Methodology Department, Real Club Celta, Vigo, Pontevedra, Spain.; 3 Department of Physical and Sports Education, Faculty of Sport Sciences and Physical Education, University of A Coruña, A Coruña, Spain.

**Keywords:** rest, fatigue, SSG, training load, game-based training

## Abstract

The aim of this study was to analyse the effects of different recovery times between bouts of small-sided games (SSG) on external and internal load variables in semi-professional soccer players. Sixteen male semi-professional soccer players performed three 4 vs. 4 + goalkeeper SSG training sessions, each with different recovery bout duration: short (1 min) (SSG1), medium (2 min) (SSG2), and long (4 min) (SSG4). Time motion and neuromuscular measures were collected during all SSGs, in addition, the rating of perceived exertion (RPE) was determined at the end of the last bout of each SSG. Results showed a significant increase in the total number of accelerations (p = 0.016, ES = 0.97, large) and decelerations (p = 0.022, ES = 0.81, large) in SSG4 compared to SSG1. In terms of the internal load, SSG2 showed significantly higher RPE values (p = 0.011, ES = 1.00, large) in comparison with SSG1. If the sessions’ focus is on neuromuscular training, longer recovery times between SSG bouts should be used. Conversely, if the aim is to reach a higher total and running distance at different intensities, a 2-min recovery period between bouts may be more appropriate.

## Introduction

Small-sided games (SSGs) are a commonly used training exercise that has gained popularity among amateur and professional soccer teams (Hill-Haas et al., 2011). SSGs represent a useful solution to improve training efficiency (Clemente et al., 2019) since they incorporate all the specific needs of soccer. One of the main advantages supporting the use of such drills allows simultaneously stimulating decision-making, physiological, physical, and technical aspects (Sarmento et al., 2018). In addition, this type of a task would allow to analyse how players manage their relations with teammates and opponents in space and time during the emergence of patterns of play at different levels (Travassos et al., 2013).

Previous scientific literature suggests that soccer and conditioning coaches can modify the characteristics of SSGs by manipulating different variables, such as the number of players (Aguiar and Botelho, 2013), pitch size (Casamichana and Castellano, 2010), the presence of the goalkeepers (Hulka et al., 2016; Radziminski et al., 2022), wildcards (Sanchez-Sanchez et al., 2017), the number of contacts allowed per player (Dellal et al., 2011), score line (Lorenzo-Martínez et al., 2020), and the duration of recovery (Köklü et al., 2015) These manipulations can influence physiological responses during SSGs, such as the heart rate, oxygen uptake, blood lactate levels, and perceived exertion (Branquinho et al., 2021a). For example, reducing the pitch size (Casamichana and Castellano, 2010) or increasing the number of players (Aguiar and Botelho, 2013) can lead to a higher intensity of exercise and increase players' physiological demands. Similarly, the inclusion of wildcards or the reduction in the number of contacts allowed per player can affect technical and tactical demands of the game, leading to different physiological responses (Dellal et al., 2011; Radziminski et al., 2016; Sanchez-Sanchez et al., 2017).

The manipulation of duration and recovery time can also affect players' physiological responses, with shorter recovery times leading to increased fatigue and decreased performance (Branquinho et al., 2020a). The modification of these factors can be broadly characterized as either continuous, where the exercise is performed without any breaks or intervals, or fractionated, where the exercise is repeated with rest periods between each repetition (Branquinho et al., 2020b). However, despite the importance of the duration of recovery during SSGs, little attention has been given to the impact of varying recovery periods between sets or repetitions on both external and internal loads in soccer players (Branquinho et al., 2021b; Köklü et al., 2015). In this regard, a recent investigation developed by Branquinho et al. (2021b) evaluated the impact of different recovery times during a 5 vs. 5 SSG format with goalkeepers (GKs). Specifically, they found that short recovery times (i.e., 30 s) resulted in a significantly greater external load compared to longer recovery times between bouts in semi-professional soccer players. Similar trends were observed for the internal load, showing greater values in SSGs with shorter recovery times between bouts. Previously, Köklu et al. (2015) analyzed the impact of different recovery times between bouts in a 4 x 4 min 3 vs. 3 SSG format without goalkeepers in youth soccer players. Their findings showed that shorter recovery times between bouts led to an increase in walking distance during SSGs. Conversely, SSGs with longer recovery times between sets resulted in more distance covered at high intensity (>18 km•h^−1^). On the contrary, Mclean et al. (2016) did not find differences between 30 s and 120 s of recovery time on external load measures in professional soccer players. However, those authors showed significant differences in heart rate values between both recovery times, with 30 s reaching higher values than 120 s. This suggests that short recovery periods may result in higher heart rate values.

As far as our knowledge goes, the effects of recovery duration during SSG formats with GKs have only been examined in one previous study. Consequently, more research is needed to determine the optimal recovery duration for subsequent SSG bout performance. Therefore, the aim of this study was to investigate the impact of different recovery duration between bouts of 4 vs. 4 SSG with goalkeepers on external and internal load variables in semi-professional soccer players. Based on earlier investigations (Branquinho et al., 2021b; McLean et al., 2016), it was hypothesized that short recovery times would increase the walking distance, low-intensity distance, and the internal load, while longer recovery times would increase moderate- and high-intensity distances.

## Methods

### Design

A randomized crossover design was applied to investigate the differences between recovery times (1 min, 2 min and 4 min) between bouts in the 4 vs. 4 + GK SSG and evaluate physical as well as physiological responses. Comparisons were performed examining male semi-professional soccer players during the 2020–2021 competitive season. The study protocol was conducted during the end of the season (i.e., May and June).

### Participants

Sixteen male semi-professional soccer players (age: 24.8 ± 6.8 years; body height: 179.1 ± 6.1 cm; body mass: 74.6 ± 7.5 kg) from two teams playing in the Spanish third division took part in this study. However, all study participants were categorized as seasoned soccer players, with 19.4 ± 4.0 years of systematic soccer training. Their regular training regimen consisted of 4 sessions per week, each lasting 90 min, and one official match on the weekend. All the players were notified of the research design and its requirements, as well as the potential benefits and possible risk associated with their participation in the study, and they signed a written informed consent document. The research procedures were approved by the Ethical Institutional Review Committee of the Faculty of Education and Sports Sciences (10–0721), in accordance with the Declaration of Helsinki.

### Procedures

Players performed three SGG training sessions (4 vs. 4 + GK) with different recovery duration: short (1 min), medium (2 min), and long (4 min) (Köklü et al., 2015). SSGs followed a 20-min standardized warm-up, which included low intensity running, dynamic stretching and a ball possession game (Sampaio et al., 2014). In each training session, only one SSG recovery period between bouts was implemented and examined. All experimental sessions were performed across the competitive season. The study sessions were carried out with more than 72 hours before or after the last match and 48 hours between them. Randomized order was applied. In the first session, a 4-min recovery (SSG4) regimen was adopted, in the second session a 2-min recovery (SSG2) regimen was used, and in the third session, 1 min of recovery (SSG1) between bouts was allowed (Köklü et al., 2015). All sessions were completed at 9:00 p.m. on artificial turf and without rain. The pitch size was 30 x 25 m (Owen et al., 2013), with a relative pitch area of 94 m^2^ per player (excluding goalkeepers) during all recovery conditions (Lorenzo-Martínez et al., 2020). The 4-a-side SSGs consisted of 4 bouts, each lasting for 4 min. To avoid potential imbalance between teams and homogenizing the competitive level, the head coach distributed players into two teams based on their level and fitness status (Clemente et al., 2019). The composition of the teams remained constant throughout the study. Similarly to previous research, teams were structured into formations of defenders, midfielders and attackers, and were allowed to interchange positions freely during the game (Coutinho et al., 2019). Before the game, players were informed of the rules, the play was restarted from the GK, and the offside rule did not apply. Several balls were distributed around the experiment performance area in order to minimize trial stoppages (Silva et al., 2015). SSGs took place with coach and conditioning specialist encouragement. Additionally, the score-line during each bout was recorded by the coaching staff of which players were aware.

### Data Collection

Data corresponding to the players’ external load during SSGs were collected using a portable 10 Hz GPS device (Playertek, Catapult Innovations, Melbourne, Australia), which incorporated a tri-axial 400-Hz accelerometer. The 10-Hz frequency is both valid and reliable for measuring the position and speed in a sports environment (Scott et al., 2016), and such devices were used in previous research with soccer players (Lorenzo-Martínez et al., 2020). Running variables obtained from the GPS were the total distance covered (m), and the distance covered (m) at four different speed thresholds (Lorenzo-Martínez et al., 2020): low-intensity running (0–6.9 km•h^−1^), medium-intensity running (7.0–12.9 km•h^−1^), high- intensity running (13.0–17.9 km•h^−1^), and sprinting (≥18.0 km•h^−1^). The total number of accelerations and decelerations was gathered (Akenhead et al., 2013). In addition, global load measures were also considered as variables: power score (w•kg^−1^), player load, work:rest ratio, and high metabolic power (HMP). Power score measures power output used per kilogram of individual’s body mass, the score is based on both the speed levels reached and the acceleration rates achieved throughout the session. Player load is a measure based on the instantaneous rate of change of tri-axial accelerometer measures (Bredt et al., 2020). The work:rest ratio represents the percentage of time that a player runs above 5.4 km•h^−1^, while the HMP is the number of efforts surpassing 20 W·kg^−1^.

The internal load of players was quantified using Foster's 0–10 scale to record the rating of perceived exertion (RPE) immediately after each repetition of the SSG (Foster et al., 2001). To prevent players from being influenced by responses of their teammates, each player rated their effort individually. Responses were written on paper and then recorded as software data. All participants were familiar with the RPE scale as they had previously employed it during regular training sessions throughout the season.

### Statistical Analysis

All statistical analyses were performed using statistical software R, version 4.2.1 (R Core Team, 2020) for Macintosh. Results are reported as means and standard deviations (mean ± SD). A linear mixed model was performed to compare the effects of different recovery times between SSGs (SSG4, 4 min of recovery; SSG2, 2 min of recovery; SSG1, 1 min of recovery) on accumulated external load variables using the R package “lme4” (Bates et al., 2015). The player identity was modeled as a random effect. Partial eta squared (ηp2) was calculated as a measure of effect size. An effect of ηp2 ≥ 0.01 indicates a small, ≥ 0.059 a medium, and ≥ 0.138 a large effect (Cohen, 1988), Furthermore, a pair-wise comparison was conducted via the Bonferroni test. The assumption of homogeneity and normal distribution of the residuals were checked graphically for each model. In addition, ES for significant pair-wise comparison was calculated using Cohen’s *d*. The magnitude of standardized mean differences was classified as trivial (< 0.2), small (0.2 ≤ *d*< 0.5), medium (0.5 ≤ *d*< 0.8), and large (*d*> 0.8) (Cohen, 1988). The homogeneity of variances was examined using the Levene’s test. For all analyses, the significance level was established at *p*< 0.05.

## Results

Table 1 displays the external and internal load effects of the different recovery times between sets during SSGs. The results showed a significant decrease in the total number of accelerations in SSG1 compared to SSG2 (*p* = 0.03, ES = 0.58, medium) and SSG4 (*p*< 0.01, ES = 0.98, large). There was also a significant decrease in the total number of decelerations in SSG1 compared to SSG2 (*p* = 0.019, ES = 0.64, medium) and SSG4 (*p*< 0.01, ES = 0.88, large). However, no significant differences (*p*> 0.05) were found for total distance and distance covered at different speed thresholds, power plays, power score, and the work-rest ratio between different recovery times. In terms of the internal load, SSG2 showed significantly higher RPE values (*p*< 0.01, ES = 1.00, large) in comparison with SSG1.

**Table 1:** Differences in the players’ internal load and external load according to the duration of recovery intervals between sets (mean ± SD).

	SSGR1	SSGR2	SSGR4	*p*-value	*ηp2*	Post-Hoc
Total Distance (m)	1750.4 ± 153.6	1789.0 ± 150.7	1731.3 ± 155.8	0.313	0.092	
Walking (m)	666.6 ± 48.2	657.5 ± 51.5	661.1 ± 49.6	0.752	0.023	
Low-intensity running (m)	701.5 ± 105.5	732.6 ± 115.3	727.7 ± 115.0	0.514	0.050	
Medium-intensity running (m)	238.8 ± 76.4	312.8 ± 74.1	268.7 ± 84.3	0.217	0.120	
High-intensity running (m)	92.5 ± 40.6	86.1 ± 34.2	73.6 ± 32.4	0.519	0.053	
Maximum Speed (km•h^−1^)	20.94 ± 1.6	20.8 ± 0.9	20.9 ± 1.3	0.789	0.020	
Total accelerations (n)	135.9 ± 17.0	146.9 ± 20.3	152.5 ± 16.7	< 0.001	0.559	SSGR1 < SSGR2^c^, SSGR4^c^
Total decelerations (n)	133.7 ± 14.0	144.7 ± 20.1	146.5 ± 15.3	0.001	0.448	SSGR1 < SSGR2^c^, SSGR4^c^
Power score (w•kg^−1^)	9.4 ± 0.9	9.6 ± 0.9	9.3 ± 1.0	0.312	0.092	
Player load	85.4 ± 9.4	88.9 ± 11.8	88.5 ± 13.4	0.124	0.160	
Work:rest ratio (%)	49.2 ± 6.0	50.9 ± 7.4	48.9 ± 7.1	0.520	0.053	
RPE	6.5 ± 0.7	7.2 ± 0.7	6.8 ± 0.6	0.008	0.334	SSGR2 > SSGR1^c^

SSGR1 = Small-sided games with 1-min recovery between sets; SSGR2 = Small-sided games with 2-min recovery between sets; SSGR4 = Small-sided games with 4-min recovery between sets; *ηp2=* partial eta squared.

a *p*< 0.05; ^b^ *p*< 0.01; ^c^ *p*< 0.001

## Discussion

This study investigated the effects of different durations between bouts of SSGs on external and internal load variables in semi-professional soccer players. Overall, findings indicated that varying the recovery periods between bouts of SSGs induced differences in neuromuscular responses and internal loads measured as perceived exertion. However, the length of the recovery period did not influence running variables.

Longer recovery periods (i.e., 4 min) led to a significant increase in both total accelerations and decelerations, whereas recovery periods of 2 min tended to increase medium and high intensity running performance, however, significant differences were not reported between these two recovery periods. The study found that SSGs with 4-min recovery periods between bouts resulted in a significantly higher number of both total accelerations and decelerations compared to SSGs with 1-min recovery periods. Nevertheless, to the best of our knowledge, there have been no previous studies that analysed the effects of different recovery periods on neuromuscular performance (accelerations and decelerations) during SSGs. Buchheit and Laursen (2013) used runners to compare 1- and 2-min rest intervals, and they stated that 2 min of recovery enabled runners to maintain higher running speeds. Phosphocreatine (PCr) plays a crucial role in supplying the energy required during intermittent activities, thus, it is probable that higher phosphocreatine resynthesis occurs during longer recovery duration (Dupont et al., 2003) as PCrresynthesis is key for high intensity actions. In consequence, short intermittent runs characterised as accelerations and decelerations are enhanced. Thus, longer recovery periods would allow players to maintain the ability to repeat high intensity explosive actions over time (i.e., accelerations and decelerations). However, longer recovery times did not result in better time-motion performance during SSGs. Similarly to the findings of Kôklü et al. (2015), distances covered at medium- and high-intensity tended to be higher during SSGs with shorter recovery duration (i.e., 2 min). This might be due to the fact that in small-pitch tasks, accelerations, decelerations, and changes of direction are more important, thus high-intensity distances are not frequently encountered throughout SSGs (Clemente et al., 2019; Jastrzębski et al., 2015). However, contrary to our results, Branquinho et al. (2021b) showed that in semi-professionals soccer players, the use of only 30 s of recovery between bouts increased both the external (distance at different velocities) and the internal load. Those authors noted that recovery periods longer than 30 s may imply a strain of mental fatigue derived from stress related to the anxiety of wanting to play for as long as possible. Differences found between both studies could be attributed to the sample used, as in our study semi-professional players, who exhibited a lower mental, physical, and technical performance during SSGs (Dellal et al., 2011), were included.

Decreasing the length of recovery periods has also negative effects on physical performance. SSGs with 1-min recovery between bouts resulted in lower total distance and running distance covered at different intensities, as well as lower neuromuscular performance. The reduction in muscle pH concentration and phosphocreatine (PCr) availability during the shorter recovery duration (i.e., 1 min) may be responsible for this decline in performance. In addition, McLean et al. (2016) found that increasing the duration of the recovery period from 30 to 120 s significantly improved physiological recovery in experienced semi-professional players, as evidenced by the increased oxygenation of the vastus lateralis muscle.

Regarding the internal load, Köklü et al. (2015) reported an increase in RPE values when recovery time decreased. In contrast with those results, our analysis revealed that RPE values were significantly higher when 2 min instead of 1 min of recovery were allowed. A possible explanation of these results is that, although non-significantly, during SSG2, a higher total distance was reported, and in accordance with previous research, the internal load was significantly correlated with total distance (Clemente, 2018; de Dios-Álvarez et al., 2021). This fact could explain the differences found in the internal load results reported in both studies.

Finally, based on our results, when the purpose of SSGs is to achieve a higher number of short high intensity activities, a longer recovery period is needed to ensure an optimal performance in explosive actions. Since during SSGs short intermittent runs characterized by acceleration and deceleration phases are frequent, the energy requirement is enhanced. Consequently, a higher level of recovery is beneficial as it allows to maintain high performance in subsequent bouts. In accordance with Buchheit and Laursen (2013), rest intervals should last at least 3 or 4 min to maintain high-intensity explosive actions. However, when the SSGs’ aim is to attain higher total and high intensity distances, then a shorter recovery period (i.e., 2 min) could be appropriate. This could have a psychophysiological justification. In accordance with Branquinho et al. (2021b), the use of long stoppages may imply strain of mental fatigue inducing boredom and lack of motivation in players, and in consequence, it can reduce muscle temperature too much (McLean et al., 2016), leading to decreased total distance covered and distance covered at high intensity.

This study has several limitations that should be taken into account. The use of only one specific format of SSG may limit the generalizability of the results. Further research incorporating different variations (i.e., modifying the number of players, pitch size, and the number of bouts) of the exercise could provide a more comprehensive understanding of the effects of altering recovery times on subsequent performance. Additionally, more different recovery times should be analyzed in order to determine how internal and external loads vary under such different conditions. The small sample size of this study may be also considered a limitation, and the conclusions drawn from the results should be approached with caution, although the number of participants was similar to other studies that examined physical and physiological responses to different training regimes. However, a larger sample of players would allow to obtain more representative values. Nevertheless, this research provides evidence about relationships between both external and internal loads and recovery duration between bouts during SSGs. These findings could help coaches and conditioning specialists optimize the design of SSGs to improve players' performance.

## Conclusions

This study highlights the significance of recovery time management during small-sided games (SSGs) to enhance the external and internal load of players. The findings indicate that semi-professional soccer players tend to exhibit more accelerations and decelerations when the recovery time between bouts is longer during SSGs. Additionally, reducing the recovery time between bouts resulted in lower RPE values.

## Practical Implications

Considering the relationship between recovery times and players' external and internal loads during SSGs, coaches and conditioning specialists can tailor their training programs to optimize players’ performance. For instance, if the focus of a training session is on neuromuscular development, longer recovery times between SSG bouts are more suitable. Conversely, if the training objective is to achieve a higher total and running distance at different intensities, 2-min recovery periods between bouts seem more appropriate.
